# Immune cell diversity and regenerative markers reveal interactions among macrophages, rodlet cells, and stem cells in the kidney of *Poecilia sphenops*

**DOI:** 10.1038/s41598-025-11679-3

**Published:** 2025-07-16

**Authors:** Ramy K. A. Sayed, Marwa Bakry, Doaa M. Mokhtar

**Affiliations:** 1 Department of Anatomy and Embryology, Faculty of Veterinary Medicine, Sohag University, Sohag, 82524 Egypt; 2 Department of Cell and Tissues, Faculty of Veterinary Medicine, Assiut University, Assiut, 71526 Egypt

**Keywords:** Macrophages, Nrf2, Rodlet cells, S100 protein, Sox9, Cell biology, Zoology, Anatomy

## Abstract

The kidneys of molly fish (*Poecilia sphenops*) exhibit complex immune and cellular activities, which are crucial for maintaining renal function and responding to environmental stressors. This study aimed to investigate the histological and immunohistochemical characteristics of immune cells, autophagy, and stem cell activity within the renal tissues of molly fish. Histological analysis revealed the presence of immune cells, including macrophages and granular leukocytes, concentrated around the renal corpuscles (RC) and renal tubules (RT). Additionally, numerous lymphocytes were observed surrounding the RC, and a notable presence of rodlet cells with a thick capsule and rodlet-like inclusions was detected around the RT. Immunohistochemical staining confirmed macrophage activity through CD68 and Iba1 expression, while APG5, an autophagy marker, was observed in macrophages, rodlet cells, and podocytes, indicating active autophagic processes. Polymorphic granulocytes expressed iNOS-2. Inflammatory markers IL-1β and NF-κB were highly expressed in rodlet cells and macrophages, respectively, suggesting their role in immune modulation. The expression of S100 protein in rodlet cells and acetylcholine in macrophages further highlights their specialized functions in immune regulation. Additionally, renal stem cells were identified by expressing Nrf2 and Sox9, indicating a potential role in tissue repair and regeneration. These findings provide critical insights into the kidney’s dual function in immunity and regeneration, contributing to a better understanding of fish renal physiology and potential applications in environmental monitoring and aquaculture health management.

**Supplementary Information:**

The online version contains supplementary material available at 10.1038/s41598-025-11679-3.

## Introduction

The kidney is a vital organ in fish, playing essential roles in osmoregulation, waste excretion, and immune defense^1^. In aquatic environments, fish are constantly exposed to various pathogens, environmental stressors, and pollutants, making the kidney a critical site for immune responses and detoxification processes^2^. The renal tissue of fish exhibits complex interactions between different cell types, including immune cells, stem cells, and specialized cells like rodlet cells. These interactions are essential for maintaining renal homeostasis and responding to external challenges^3^.

Previous studies have highlighted the importance of macrophages, a key component of the innate immune system, in mediating immune responses within the kidney^4^. Macrophages are involved in phagocytosis, antigen presentation, and the production of cytokines that orchestrate inflammatory responses^5^. The macrophages are characterized by their plasticity which can assume different functional states depending on the signals they receive from their environment. For instance, in the presence of pathogens or tissue damage, macrophages can adopt an activated state, producing pro-inflammatory cytokines such as IL-1β, which are critical for initiating and sustaining immune responses^6^. Additionally, macrophages in fish are involved in tissue repair and autophagy, a cellular process that helps remove damaged organelles and pathogens from within cells^7^.

Rodlet cells are unique to fish and have intrigued researchers due to their specialized structure and enigmatic function. These cells are found in a variety of tissues, including the kidney, gills, and skin, which are primary interfaces between the fish and its environment. Rodlet cells are characterized by their thick capsule and rod-shaped inclusions (or “rodlets”). Their exact role in fish immune responses is still debated; however, several hypotheses suggest that rodlet cells are involved in host defense, particularly in response to parasitic infections and environmental stressors^8^.

Molly fish (*Poecilia sphenops*) are widely used as model organisms in aquatic research due to their adaptability to diverse environmental conditions^9,10^ and their relevance in studying physiological and immunological processes^11^. Additionally, the molly fish is recognized as a valuable animal model in various studies^12–17^.

This study focuses on exploring the histological and immunohistochemical features of immune and stem cells in the kidneys of molly fish. The present work provides valuable insight into the distribution and activity of key immune cells such as macrophages and rodlet cells, as well as the expression of autophagy and inflammatory markers. The findings contribute to the broader knowledge of how fish kidneys manage immune responses and adapt to environmental stressors, shedding light on the dynamic interactions within renal tissues.

## Results

### Histological analysis

**Fig. 1:**
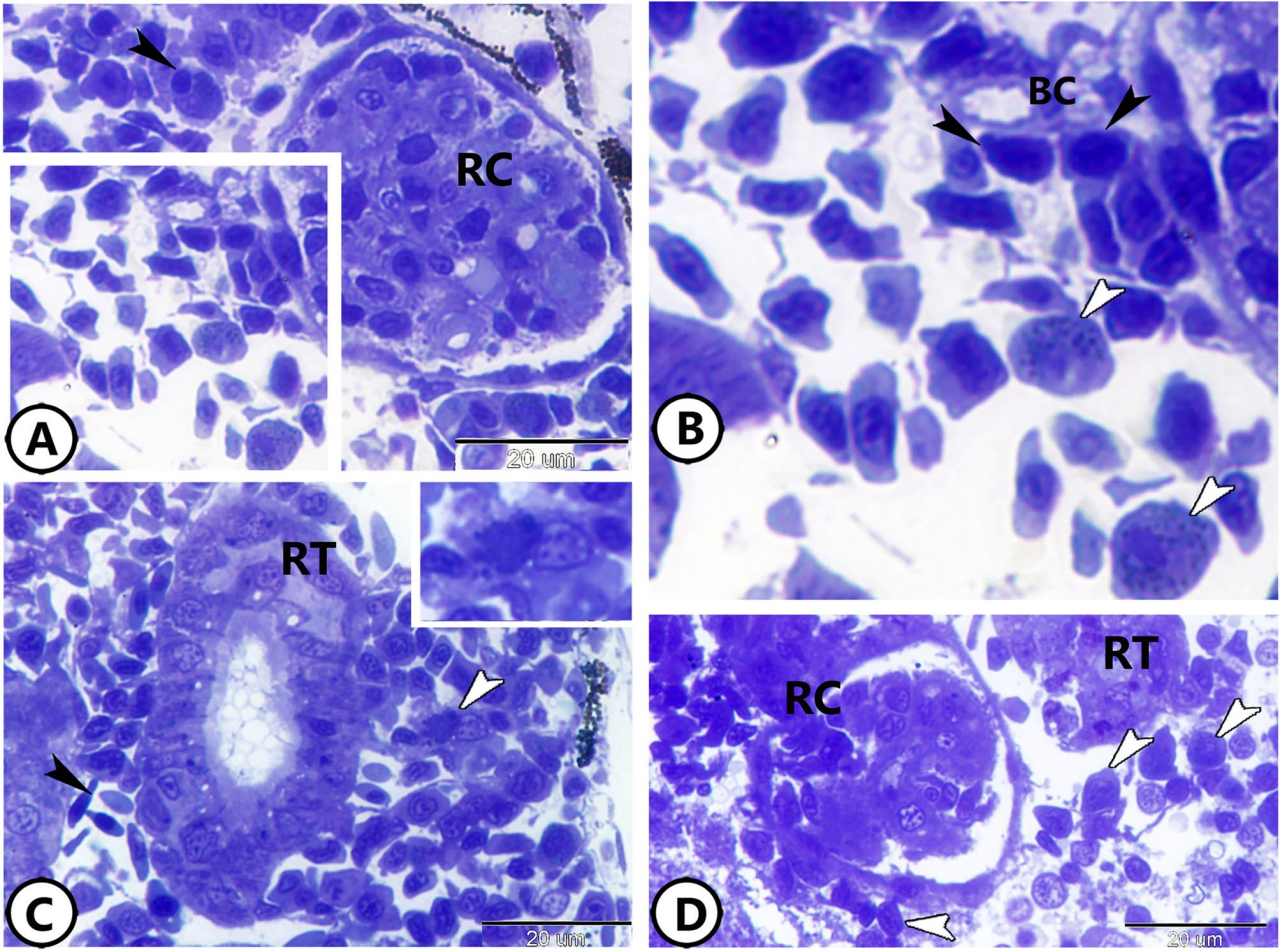
Semithin section of the kidney of the molly fish stained with toluidine blue. (**A**) The renal corpuscle (RC) is surrounded by neutrophils (boxed area) and macrophages (black arrowhead). (**B**) Higher magnification of the neutrophils (white arrowheads). Note the presence of lymphocytes (black arrowheads) around the blood capillaries (BC). (**C**) Renal tubules (RT) with many RBCs (black arrowhead) and macrophages (white arrowhead, boxed area). (**D**) The renal corpuscle (RC) is surrounded by many lymphocytes (arrowheads).

Toluidine blue staining of semithin sections revealed significant immune cell presence and activity within the kidneys of molly fish. The renal corpuscle (RC) was frequently surrounded by neutrophils and macrophages (Fig. 1A). The macrophages showed eccentric nuclei and dark cytoplasm contained phagocytic materials (Fig. 1A). At the same time, the neutrophils were characterized by the presence of metachromatic granules and lobulated nuclei (Fig. 1B). Renal tubules (RT) were also surrounded by macrophages (Fig. 1C). Additionally, numerous lymphocytes were observed encircling the RC (Fig. 1D).

**Fig. 2:**
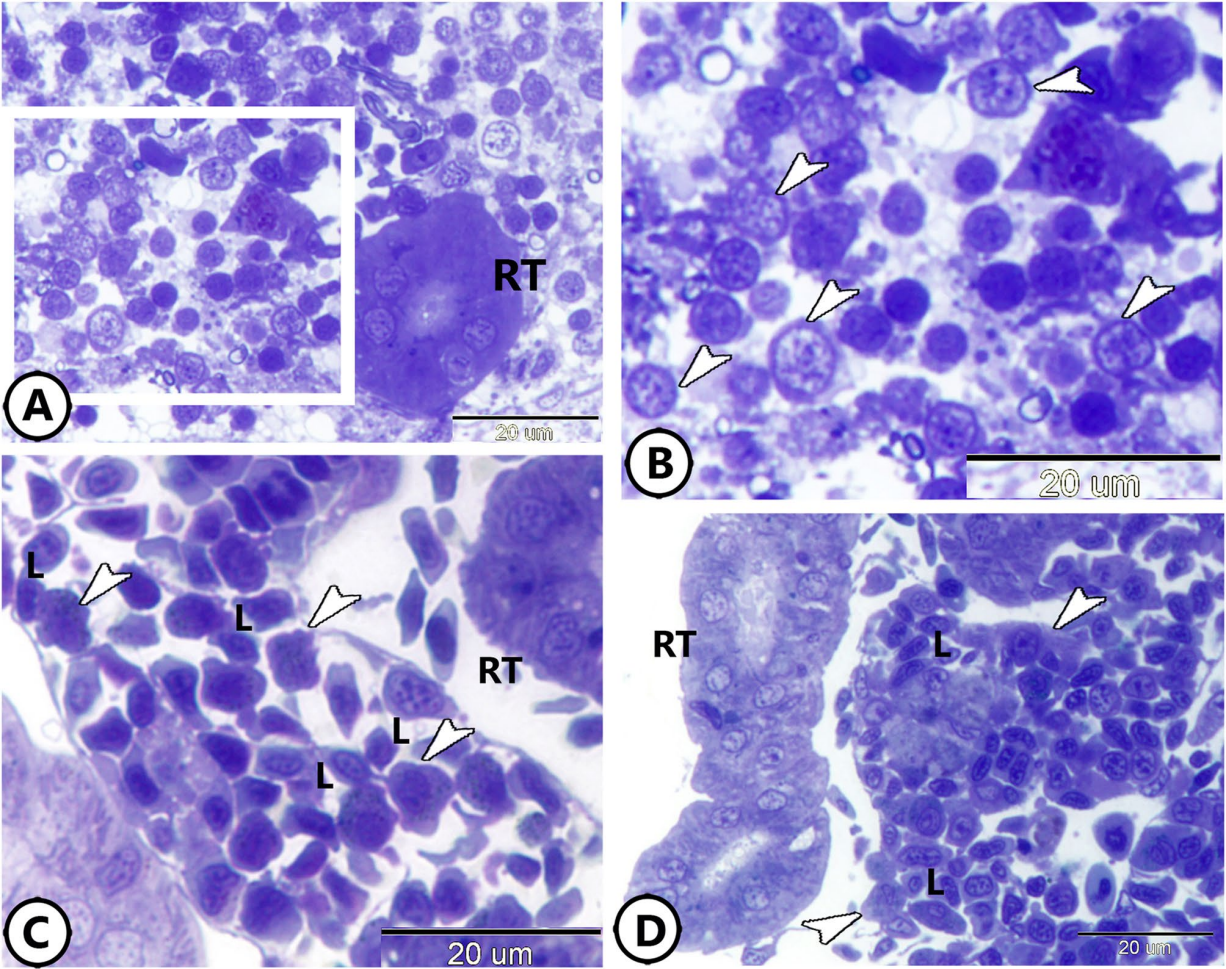
Semithin section of the kidney of the molly fish stained with toluidine blue. (**A**,**B**) Low and higher magnification show the distribution of rodlet cells (arrowheads) around the renal tubules (RT). (**C**,**D**) Colocalization of lymphocytes (L) and macrophages (arrowheads) around renal tubules (RT).

Furthermore, a notable presence of rodlet cells was observed around the RT (Fig. 2A, B). The rodlet cell consisted of a thick capsule and rodlet-like inclusions (Fig. 2B). The colocalization of macrophages and lymphocytes was evident around the renal tubules (Fig. 2C, D).

### Immunohistochemical analysis

**Fig. 3:**
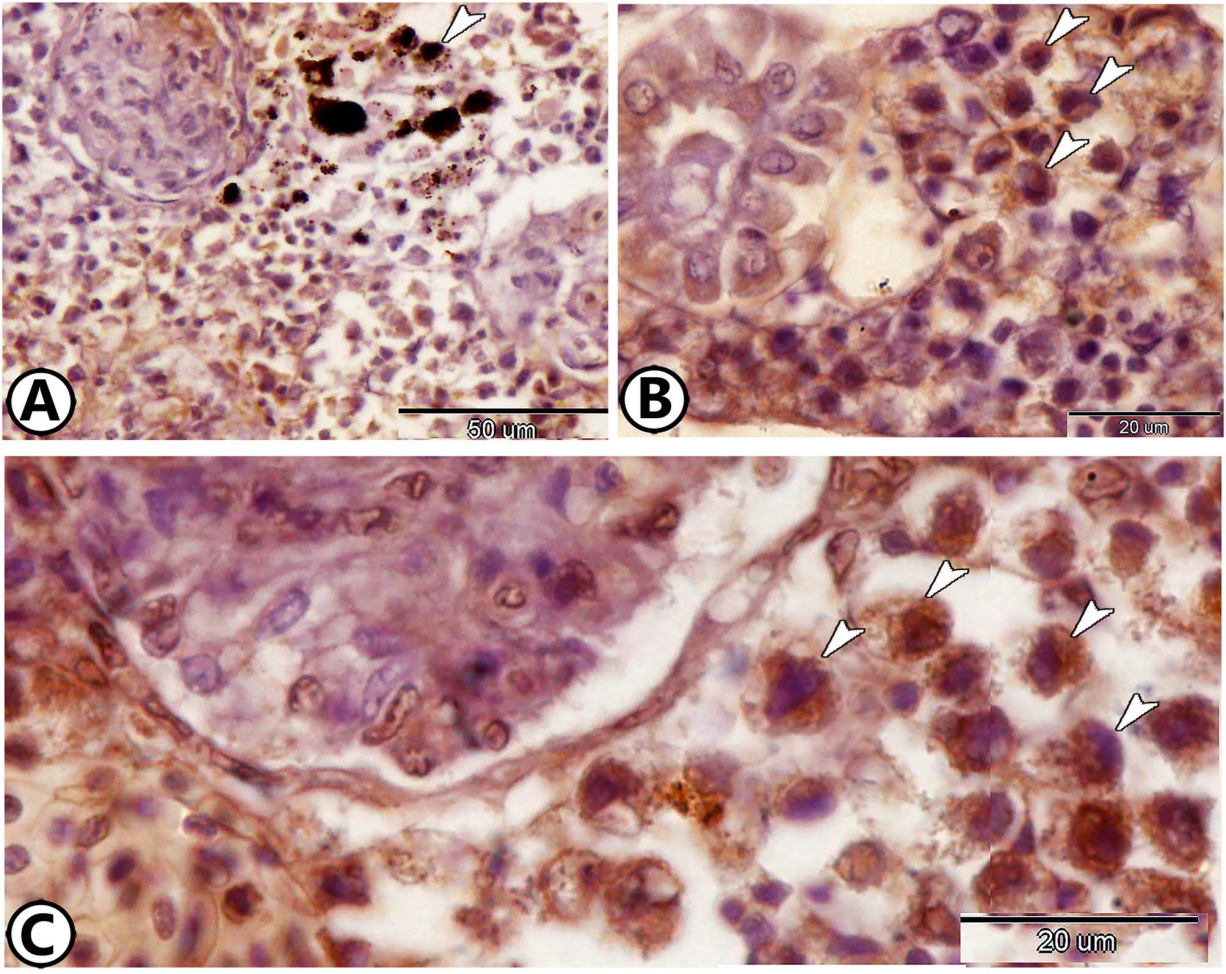
Immunohistochemistry of CD68 and Iba1. (**A**) The melanomacrophage center (arrowhead) expresses CD68. (**B**) Macrophages (arrowheads) express CD68. (**C**) Macrophages (arrowheads) express Iba1.

Immunohistochemical staining for CD68 and Iba1 revealed distinct macrophage activity within the renal tissue. CD68 expression was prominent in the melanomacrophage centers (Fig. 3A). Additional CD68-positive macrophages were scattered throughout the renal tissue (Fig. 3B). Iba1 staining further confirmed macrophage presence and activity (Fig. 3C).

**Fig. 4:**
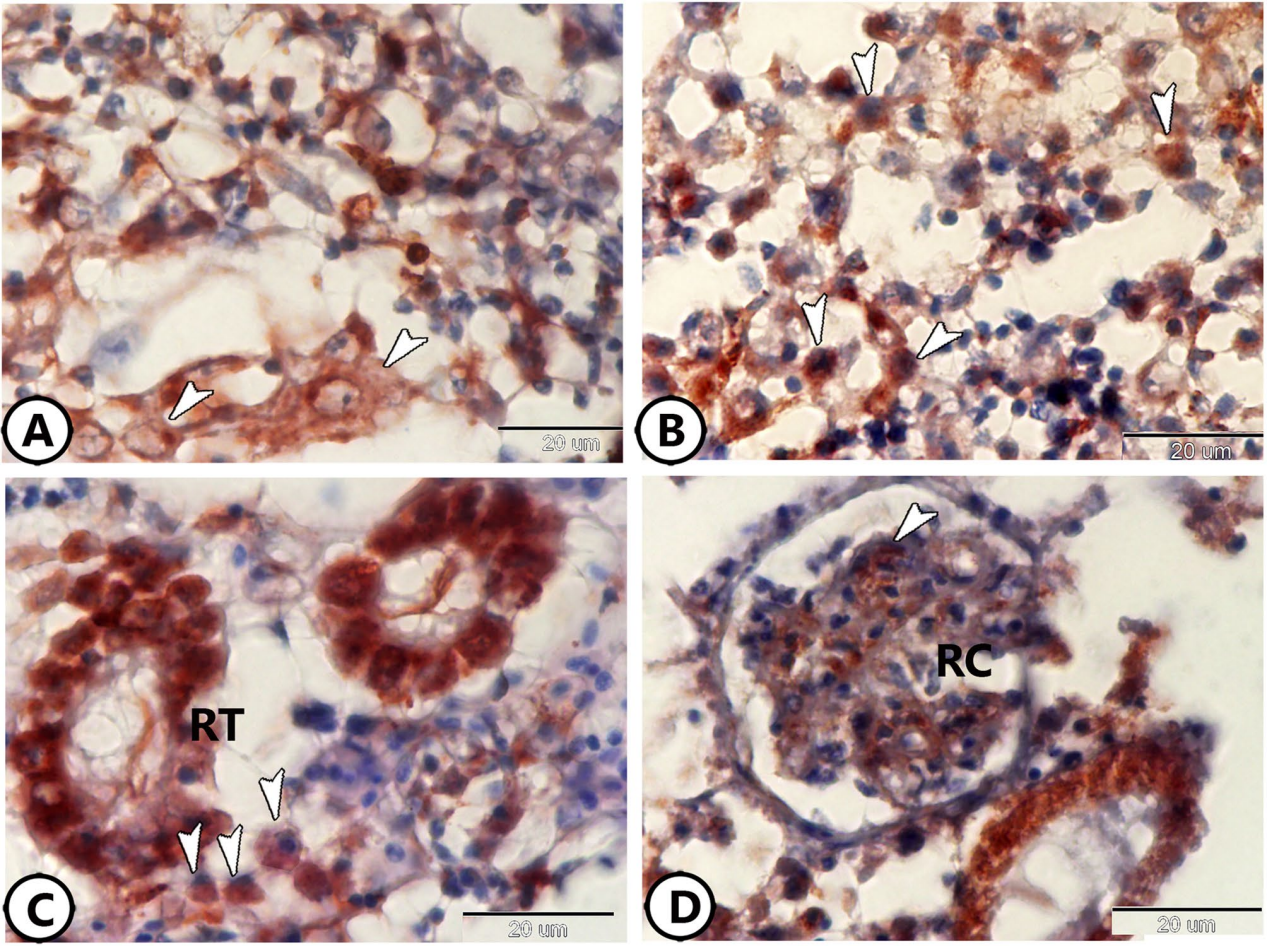
Immunohistochemistry of APG5. (**A**,**B**) Macrophages (arrowheads) express APG5. (**C**) Rodlet cells (arrowheads) express APG5 around renal tubules (RT). (**D**) Podocytes (arrowheads) express APG5 in the renal corpuscle (RC).

Immunostaining for APG5, a marker of autophagy, demonstrated significant autophagic activity within renal immune cells. Macrophages expressing APG5 were widespread throughout the renal tissues and were characterized by amoeboid-shaped cell bodies with cell processes (Fig. 4A, B). Additionally, rodlet cells with excentric nuclei surrounding the RT exhibited strong APG5 cytoplasmic expression (Fig. 4C). Podocytes within the RC also expressed APG5 (Fig. 4D).

**Fig. 5:**
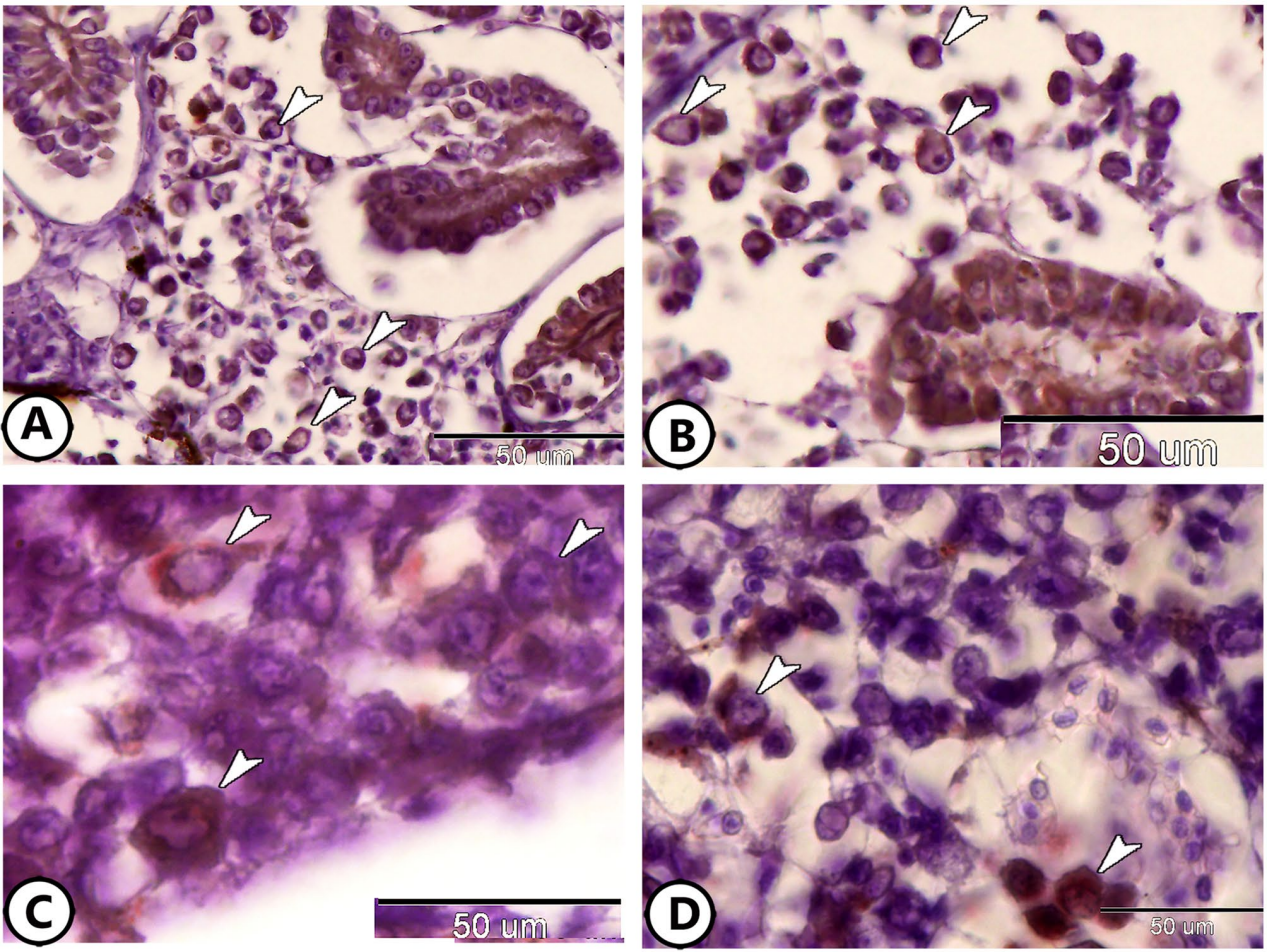
Immunohistochemistry of IL-1β and NF-κB. (**A**,**B**) Rodlet cells (arrowheads) express IL-1β around renal tubules. (**C**,**D**) Macrophages (arrowheads) express NF-κB.

The inflammatory markers (IL-1β and NF-κB) expression was assessed to understand their role in renal immune responses. Rodlet cells surrounding the RT exhibited IL-1β expression. Rodlet cells showed eccentric nuclei and well-defined capsules (Fig. 5A, B). Macrophages also showed NF-κB expression (Fig. 5C, D).

**Fig. 6:**
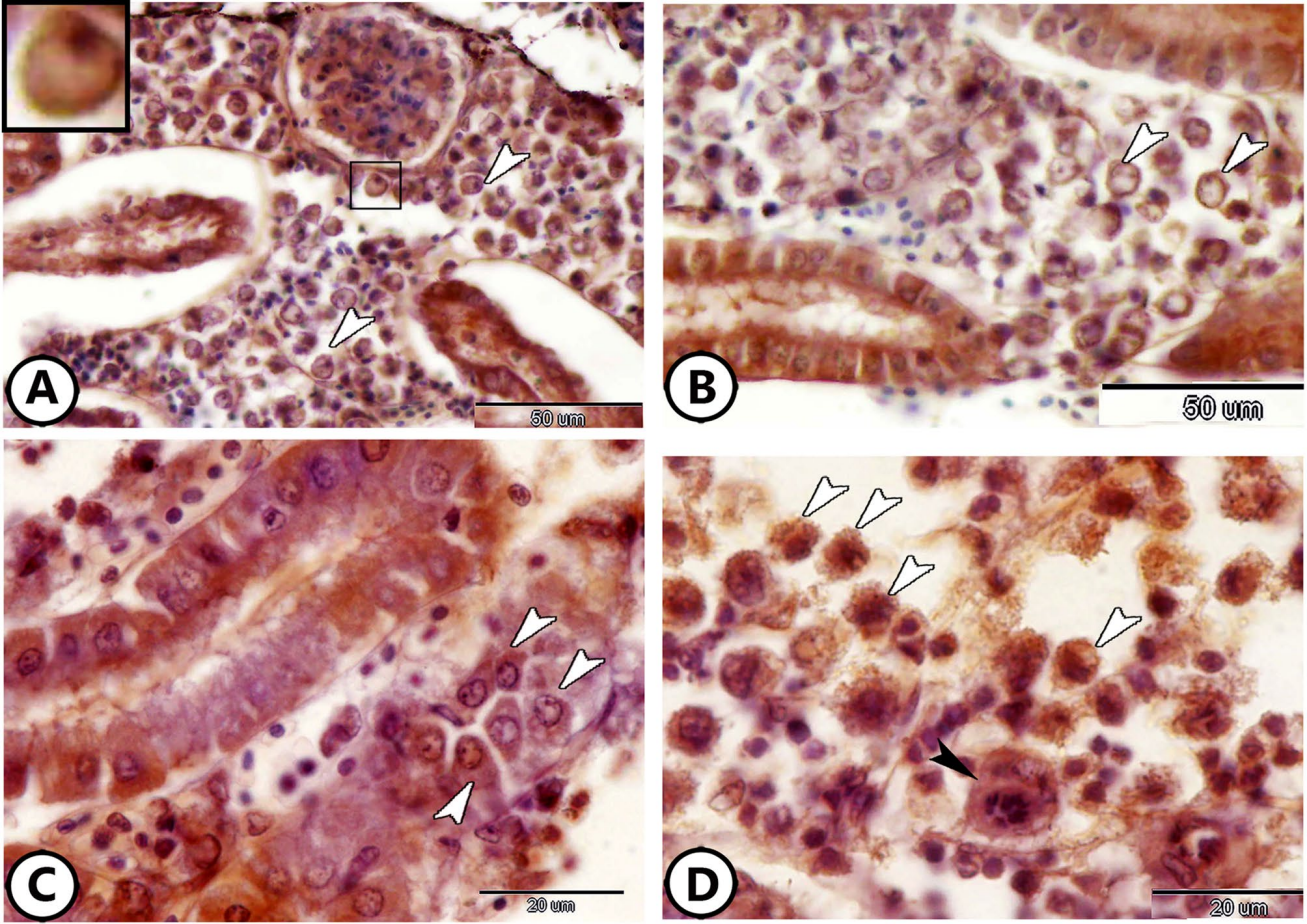
Immunohistochemistry of S100 protein and Ach. (**A**,**B**) Rodlet cells (arrowheads) express S100 protein. (**C**,**D**) Macrophages (white arrowheads) express Ach. Note the presence of dividing cells (black arrowhead).

**Fig. 7:**
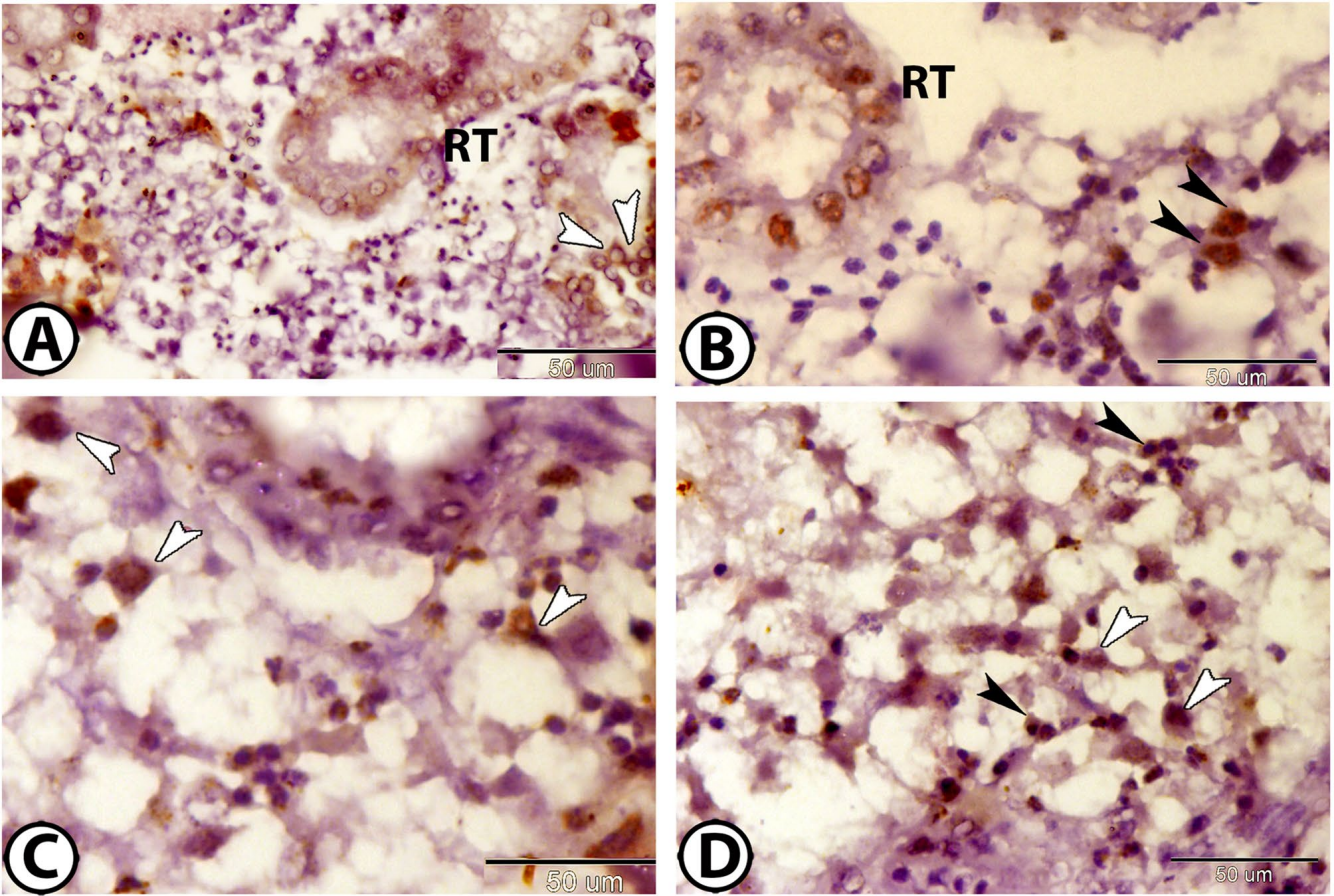
Immunohistochemistry of iNOS-2. (**A**) Rodlet cells (arrowheads) around the renal tubules (RT) expressed iNOS-2. (**B**–**D**) Polymorphic granulocytes (black arrowheads) and macrophages (white arrowheads) around the renal tubules (RT) expressed iNOS-2.

Immunostaining for S100 protein revealed its expression in rodlet cells within the renal tissue (Fig. 6A, B). Additionally, macrophages were found to express acetylcholine (Ach) (Fig. 6C, D). iNOS-2 was expressed in the capsules of rodlet cells (Fig. 7A), polymorphic granulocytes around renal tubules (Fig. 7B–D), and in the macrophages (Fig. 7C, D).

**Fig. 8:**
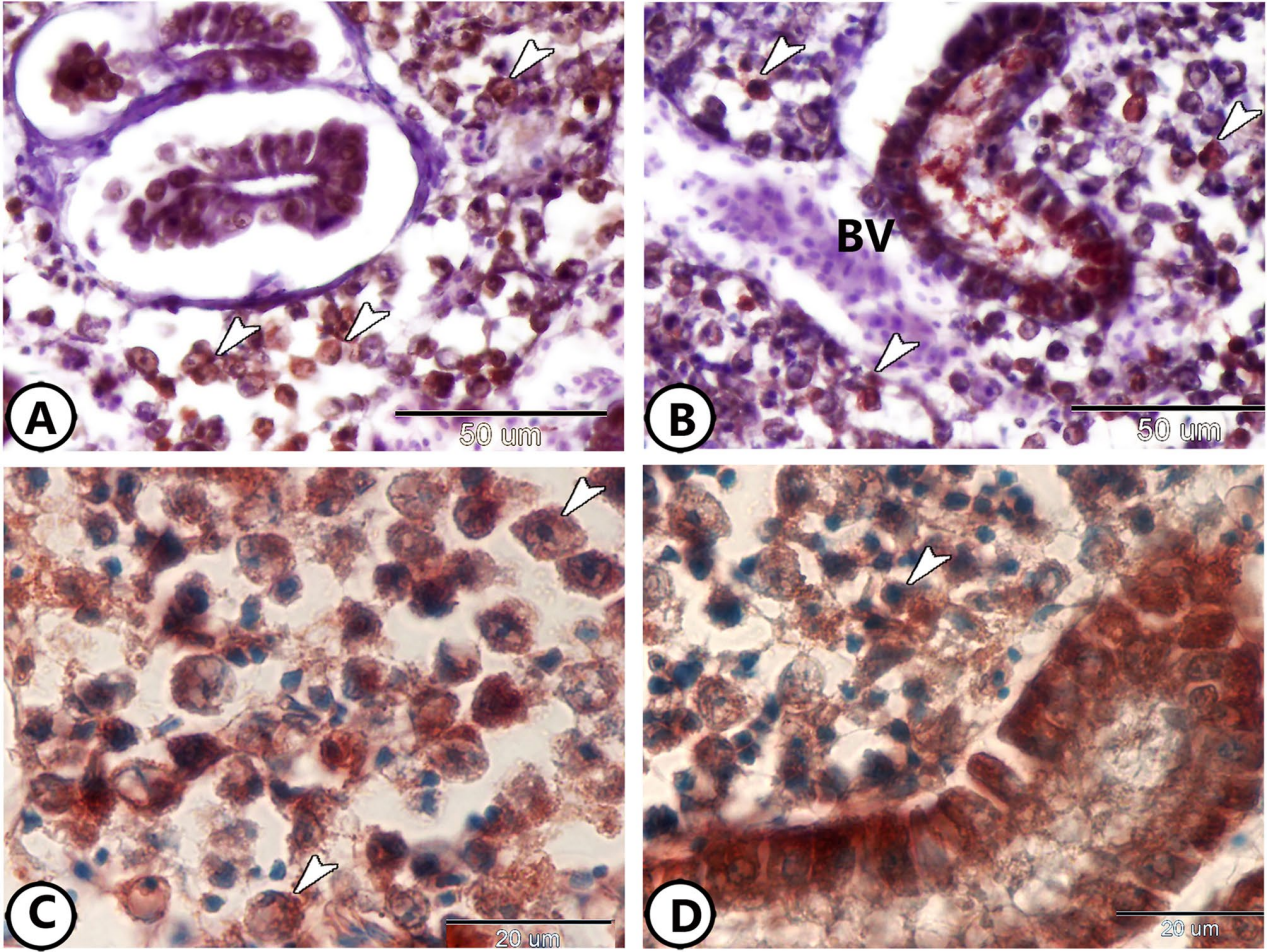
Immunohistochemistry of Nrf2 and Sox9. (**A**,**B**) Stem cells (arrowheads) around the blood vessels (BV) expressed Nrf2. (**C**,**D**) Stem cells (arrowheads) expressed Sox9.

Stem cells within the renal tissue were identified by their expression of Nrf2 and Sox9. Nrf2 expression in stem cells (Fig. 8A, B) was well distinct in the renal tissues. Additionally, the expression of Sox9 was observed in these stem cells (Fig. 8C, D).

### Quantitative Cell analysis

In addition to qualitative observations, a semi-quantitative analysis was conducted to estimate the average number of specific cell types per high-power field (HPF) at 400× magnification using light microscopy. The analysis included multiple fields from toluidine blue-stained semithin sections and immunohistochemically stained sections. The mean cell counts are summarized in (Table 1).

**Table 1:** Estimated mean number of cells per high-power field (HPF) in Molly fish kidney.

Cell type/marker	Location	Estimated mean no. per HPF	Remarks
Macrophages (CD68, Iba1)	Around RC and RT, melanomacrophage centers	7.0 ± 0.4	Present as individual cells and in clusters
Neutrophils	Around RC	4.0 ± 0.3	Identified by lobulated nuclei and granules
Lymphocytes	Surrounding RC, RT	11.0 ± 0.5	Described as “numerous”, associated with immune niches
Rodlet cells	Around RT	5.0 ± 0.3	Thick capsule; counted by toluidine blue and IHC
Granulocytes (iNOS-2)	Around RT	4.5 ± 0.3	Polymorphic morphology noted
Stem cells (Nrf2, Sox9)	Perivascular regions	3.0 ± 0.2	Small, round nuclei with marker positivity

## Discussion

The kidney of molly fish demonstrated significant infiltration and activity of various immune cells, such as granular leucocytes and macrophages, where these cells were observed around the RC and RT. Macrophages play a critical role in specific immune responses due to their involvement in lymphocyte activation and phagocytosis. The surface of these cells presents several receptors, such as toll-like receptors, pattern recognition receptors, and C-type lectin receptors, and these receptors are essential for the immune response to pathogens^18^. Furthermore, macrophages, besides their importance for antigen presentation to T cells, are considered a pivotal source of chemokines and cytokines that drive an efficient immune response^18,19^.

In teleosts, the M1 activation state “pro-inflammatory macrophage” is the best-characterized macrophage phenotype that plays a fundamental role in the protection of the host. These cells have been reported to kill pathogens rapidly through a process including engulfment and toxic reactive intermediates production, phagolysosomal acidification, and restriction of nutrient availability^20–22^. In addition, M1 macrophages are vigorous factories of lipid mediators, chemokines, and cytokines, which act to boost the inflammatory and adaptive immune responses^23^.

In molly fish, CD68 expression was predominantly observed in the melanomacrophage centers, with additional CD68-positive macrophages dispersed throughout the renal tissue. The melanomacrophage centers were previously detected in the kidneys of the grass carp^24^. These centers are implicated in the process of antigen processing and presentation to lymphocytes^25^. CD68 expression was previously detected in atrial macrophages of the molly fish^26^. It is a myeloid-specific surface marker, which is widely expressed in myeloid dendritic cells and macrophages^27^. It is primarily associated with the endosomal/lysosomal compartment, with its late endosome localization, proposing a function in peptide transport and antigen processing^28^. Furthermore, CD68-expressing macrophages are recognized as key players in the foreign body reaction^29^.

Iba1 staining provided additional confirmation of macrophage presence and activity in the renal tissue of molly fish. Iba1 expression was detected in various tissues of the molly fish such as the atrium, brain, and ovaries^16,26,30^. Iba1 expression was observed in macrophages, where it plays a vital role in facilitating membrane ruffling mediated by macrophage colony-stimulating factor^31,32^. Additionally, Iba1 is implicated in calcium signaling pathways, underscoring its importance in the processes regulating membrane ruffling and phagocytosis in macrophages^31^.

Additionally, the macrophages within the molly fish renal tissue revealed Ach expression. Ach expression was observed in the atrial rodlet cells and ventricular myocardial cells of the molly fish^26,30^. The release of Ach from efferent fibers of the vagus nerve is well-known to suppress various pro-inflammatory cytokines secretion via interacting with Ach receptors on immune cells and macrophages^33,34^. Moreover, activation of Ach has been widely reported to reduce inflammatory responses in peripheral tissues and restore impaired immune cell function^35^. In this regard, mice deficient in Ach have demonstrated an impaired cholinergic system, unable to effectively suppress inflammatory responses^36^.

Lymphocytes were observed in the kidney of the molly fish around RC. B lymphocytes, as professional antigen-presenting cells (APCs), play a fundamental role in humoral immunity by producing antibodies against antigens. They also contribute to immunological memory through their memory B cell differentiation^37^. In fish, the head kidney serves as a key site for the production and maturation of naive B cells^38,39^. To date, three distinct immunoglobulin isotypes, IgM, IgD, and IgT/Z have been identified in teleost fish^40,41^.

Rodlet cells were identified around the RTs of the molly fish, playing a key role in the fish’s defense system. These cells are widely distributed across various tissues, including the thymus, kidney, heart, gills, spleen, liver, gall bladder, skin, pancreas, and blood vessel endothelium^42,43^. They have been reported to perform several functions, notably a secretory role associated with defense, particularly against parasitic helminths^18,44^. Additionally, rodlet cells have been reported to be activated in response to tissue damage, resembling leukocyte reactions to different chemotactic stimuli^45^.

Rodlet cells, observed around the renal tubules, have long intrigued researchers due to their unique morphology and enigmatic function. While the present study identified cells expressing IL-1β, iNOS-2, and S100 protein, suggesting roles in immune modulation, an ongoing debate remains regarding their precise biological function. Some studies propose a role in innate immunity and secretion of defensive molecules, especially in response to parasitic infections or tissue injury^46,47^. Others, however, argue that rodlet cells represent a specialized form of epithelial or secretory cell with functions not strictly confined to immunity^48^. Their widespread distribution across different fish tissues further complicates a singular functional interpretation. The expression of the S100 protein by rodlet cells was detected in a wide diversity of molly fish tissues^16,26,49–51^. S100 proteins are essential components of the defense systems across various species, functioning in both extracellular and intracellular contexts. Intracellular S100 isoforms play a pivotal role in the innate immune response, contributing to pro-inflammatory stimulation, transcription regulation, cytoskeletal rearrangement, and free radical scavenging. They are crucial for regulating inflammation and treating autoimmune diseases, where they are also involved in the regulation of numerous cellular processes^52–54^. Therefore, our findings support an immunological involvement of rodlet cells in the kidney.

The expressions of IL-1β and NF-κB were detected in the rodlet cells and macrophages, respectively, suggesting the role of renal immune cells in the molly fish renal immune responses. Our previous studies identified the expression of IL-1β and NF-κB in the pancreas, spleen, atrium, intestinal bulb, ependymal cells, and liver of the molly fish^16,17,26,49,50^. IL-1β plays a diverse role in physiological functions, with fish retaining its capacity to regulate inflammatory processes^55^. It is central to initiating both local and systemic responses to various stimuli by engaging T and B lymphocytes, natural killer cells, and activating macrophages^56^. Additionally, elevated IL-1β production is involved in different cellular activities, including cell proliferation and differentiation^57^. NF-κB is an essential transcription factor in the innate immune response. This factor mediates the production of numerous pro-inflammatory cytokines and plays a vital role in various signaling pathways^58^. It plays a crucial role in responding to inflammatory and immune stimuli, regulating cell proliferation, adhesion, invasion, and apoptosis across various cell types^59^.

Autophagic activity was noted among renal immune cells, as shown by APG5 expression, where APG5 expression was detected in macrophages scattered throughout the renal tissue, rodlet cells surrounding the RT, and podocytes within the RC. APG5 is a key regulator in the autophagy process, playing a crucial role in the formation of autophagic vesicles, lymphocyte development and proliferation, apoptosis, and adipocyte differentiation^60^. Its expression was detected in different tissues of the molly fish^16,17,49–51,61^. Thus, the expression of macrophages within the renal tissue for APG5 suggests that renal immune cells are involved in the autophagy process and support the potential role of the kidney in lymphocyte development and proliferation.

The present study showed that the polymorphic granulocytes express iNOS_2_ in the kidney of molly fish. The expression of iNOS in granulocytes increases during stressing and inflammatory events, produces a cytotoxic environment, and supports proinflammatory reactions. Immunoreactivity of iNOS was also detected in leukocyte populations in the primary lymphohematopoietic organs, gills, and gut, as well as in vascular structures and MMCs of the spleen (Campos-Pérez, et al., 2000).

Stem cells within the renal tissue of the molly fish were characterized by their distinct expression of Nrf2 and Sox9. In fish, Nrf2 has a critical function in the regulation of lipid metabolism and inflammatory responses^62^. It has been shown to contribute to antioxidation, immune enhancement, and osmoregulation, alongside its well-known roles in managing toxicity and oxidative stress, especially if fish are experiencing salinity stress^63,64^. The SOX family is essential for stem cell maintenance, embryonic development, and lineage commitment. In adult tissues, particularly, Sox9 regulates stem and progenitor cells^65,66^. Moreover, Sox9 plays an essential role in regulating cell proliferation and determining cell fate during embryogenesis^67^. In molly fish, most of the studied tissues revealed the expression of Nrf2 and Sox9^16,17,26,30,49–51,61,68,69^.

In teleost fish, the kidney, particularly the anterior (head) region, is the principal site of hematopoiesis, functioning analogously to bone marrow in mammals^70^. While our study focused on the trunk kidney of *Poecilia sphenops*, the presence of lymphocytes, macrophages, and stem cell markers (Nrf2 and Sox9) suggests active immune and regenerative roles that are consistent with findings in other teleosts such as *Danio rerio*^71^ *Carassius auratus*^72^ and *Cyprinus carpio*^73^. In contrast, the trunk kidney is more traditionally associated with excretory functions, yet increasing evidence, supported by the current findings in *Poecilia sphenops*, suggests that immune and regenerative activity also persists in this region. However, some species exhibit species-specific differences in the distribution and activity of hematopoietic tissues. For example, in *Oreochromis niloticus*, melano-macrophage centers are prominent in the head kidney and closely associated with erythropoietic and lymphopoietic niches^38^. In *Cyprinus carpio*, the head kidney displays compartmentalization of granulopoiesis and lymphopoiesis^74^. In *Labeo rohita* (rohu), erythroid islands are more prominent in the head kidney, while granulocyte maturation occurs in both renal regions^75^. In contrast, *Poecilia reticulata* (guppy), a close relative of *P. sphenops*, displays MMC activity and lymphocyte accumulation even in the mesonephric trunk kidney, hinting at a possible evolutionary trend among poeciliids toward distributed renal immune functionality^76^. These features are mirrored in *Oncorhynchus mykiss* (rainbow trout), where leukocyte proliferation can occur in both kidney regions^77^. Moreover, the presence of melanomacrophage centers (MMCs) in the trunk kidney of *P. sphenops*, as shown by CD68 staining, parallels similar structures found in the kidneys of *Ctenopharyngodon idella*^24^. Further comparative and functional studies are needed to delineate how hematopoietic activity is partitioned between head and trunk kidney regions across teleost species.

The present study provides detailed anatomical and immunohistochemical insights into the presence and localization of immune and stem cell markers within the renal tissue of *Poecilia sphenops*. Future studies incorporating controlled experimental models, such as exposure to environmental toxins, salinity fluctuations, or immune challenges, will be essential to validate the proposed roles of macrophages, rodlet cells, and renal stem cells in renal adaptation. These models could help to quantify the upregulation or suppression of specific markers (e.g., IL-1β, NF-κB, APG5, Sox9, Nrf2) and confirm their involvement in stress-mediated immune or regenerative processes. Incorporating such functional assays would significantly enhance our understanding of the kidney’s adaptive capacity in teleost fish.

## Conclusion

The study findings revealed the presence of CD68, Iba1, and Ach in macrophages scattered throughout the renal tissue, indicating macrophage activity. Furthermore, the expression of autophagic (APG5) and inflammatory (IL-1β and NF-κB) proteins was identified in macrophages, rodlet cells, and podocytes within the renal corpuscles. Polymorphic granulocytes expressed iNOS-2. Nrf2 and Sox9 expression was also detected in stem cells within the renal tissue. These results highlight the complex interplay between immune responses and autophagy in the kidneys of molly fish, emphasizing the contributions of immune and stem cells to renal health and environmental adaptation. However, further research is needed to confirm these findings and investigate the effects of studied protein knockout on renal function and regenerative capacity.

## Materials and methods

The current work was performed in compliance with university animal care rules and Egyptian laws, following the ARRIVE guidelines. Every procedure used in this study has been authorized by the National Ethical Committee of the Faculty of Veterinary Medicine at Assiut University in Egypt. Aun/vet/4/0015 is the Ethical No. Al methods were performed in accordance with the relevant guidelines and regulations.

### Sample collection

The study’s materials included 16 mature healthy male molly fish (*Poecilia sphenops*, Valenciennes 1846) that were chosen at random from an ornamental fish store. On average, the specimens weighed 9.50 ± 1.20 g and measured 4.10 ± 3.0 cm in standard length. Following arrival at the laboratory, the fish were kept for an adaptation period of 2 weeks to check their health condition, and fish that exhibited abnormal appearance and/or behavior were excluded from the study. The selected fish were euthanized with an overdose of MS-222 (3% tricaine) prior to tissue sampling^78^.

### Semithin sections

Small trunk kidney specimens were initially fixed in a 2.5% paraformaldehyde-glutaraldehyde solution and left for 12 h for proper fixation. The samples were then rinsed in 0.1 M phosphate buffer and osmicated using 1% osmium tetroxide in 0.1 M sodium-cacodylate buffer (pH 7.3). Following this, the samples were embedded in Araldite after being dehydrated with ethanol and propylene oxide. Toluidine blue was applied to semithin (1 μm thick) sections, which were then seen under a light microscope.

### Immunohistochemical analysis

For immunohistochemical investigation, trunk kidney tissue sections were prepared using the UltraTek HRP Anti-Polyvalent (DAB) Staining System (ScyTek Laboratories, Utah, US, AMF080). After deparaffinizing the sections in xylene, they were rehydrated through a series of graded ethanol solutions and washed in distilled water. The sections were heated for 10 min in a sodium citrate buffer (0.01 M, pH 6.0) to increase epitope exposure. They were then left to cool at room temperature for 30 min before being cleaned with PBS. The sections were treated with 3% H_2_O_2_ in distilled water for 15 min at room temperature, and then they were washed twice in PBS for 5 min each to block endogenous peroxidase activity.

Finally, the sections were treated with the superblock solution provided in the kit for 5 min at room temperature to prevent non-specific binding. The sections were exposed to diluted (1:100) primary antibodies against the rabbit polyclonal S100 protein for an entire night at 4 °C (Z0311, Dako, Glostrup, Denmark), mouse monoclonal anti-CD68 (Santa Cruz, sc-17832), rabbit polyclonal Nicotinic Acetylcholine R alpha 7 NACHRA7 (ABclonal, A7844), rabbit polyclonal interleukin 1 beta (IL-1β) (sc-7884, Santa Cruz Biotechnology, Heidelberg Germany), rabbit polyclonal iNOS-2 (RB-1605, Thermo Fisher Scientific, UK), mouse monoclonal autophagy protein 5 (APG5) (sc-133158, Santa Cruz Biotechnology, Heidelberg, Germany), rabbit polyclonal nuclear factor kappa B (NF-κB) (10745-1-AP, Proteintech, USA), rabbit polyclonal nuclear factor erythroid 2-related factor 2 (Nrf2) (sc-722, Santa Cruz Biotechnology, Heidelberg, Germany), and rabbit polyclonal SRY-Box transcription factor 9 (Sox9) (AB5535, Sigma-Aldrich, Madrid, Spain). These antibodies were previously tested in molly fish^16,69^. Simultaneously, tissue sections that were incubated with buffer instead of S100 protein primary antibody were used as negative controls (Fig. S1A, B). Using a biotinylated secondary antibody UltraTek Anti-Polyvalent antibody that was acquired from Scy Tek (USA), sections were rinsed three times for five minutes each with PBS before being treated for fifteen minutes. After that, the tissues were incubated with the UltraTek HRP for 15 min, and the slides were washed three times for three minutes each with a wash buffer. Following the manufacturer’s instructions, the reaction was visualized using Diaminobenzodiazibin (DAB) chromogen diluted with DAB substrate (included in the same Scy Tek Detection kit) for 10 to 15 min, or until the desired staining was obtained. Harris hematoxylin was then used as a counterstain, and the reaction was mounted using DPX mounting media.

### Quantitative cell estimation

For semi-quantitative analysis, representative fields (*n* = 10 per sample) were selected from toluidine blue-stained semithin sections and immunohistochemically stained sections. Cell types, including macrophages, lymphocytes, rodlet cells, neutrophils, granulocytes, and renal stem cells, were identified per 100 µm2 using established morphological criteria and confirmed by immunolabeling. Counts were performed using ImageJ Software under 400× magnification, and the mean number of each cell type per high-power field (HPF) was calculated *±* standard error (SE).

## Electronic supplementary material

Below is the link to the electronic supplementary material.

Supplementary Material 1

## Data Availability

The datasets used and/or analyzed during the current study are available from the corresponding author upon reasonable request.
