# Supplementary material for: Immune cell diversity and regenerative markers reveal interactions among macrophages, rodlet cells, and stem cells in the kidney of Poecilia sphenops
Source: Sci Rep. 2025 Jul 16;15:25879. doi: 10.1038/s41598-025-11679-3 (PMC12267737; doi:10.1038/s41598-025-11679-3)
Supplement: Supplementary file 1 — Supplementary Material 1 [file 41598_2025_11679_MOESM1_ESM.docx]

**Supplementary Figures**


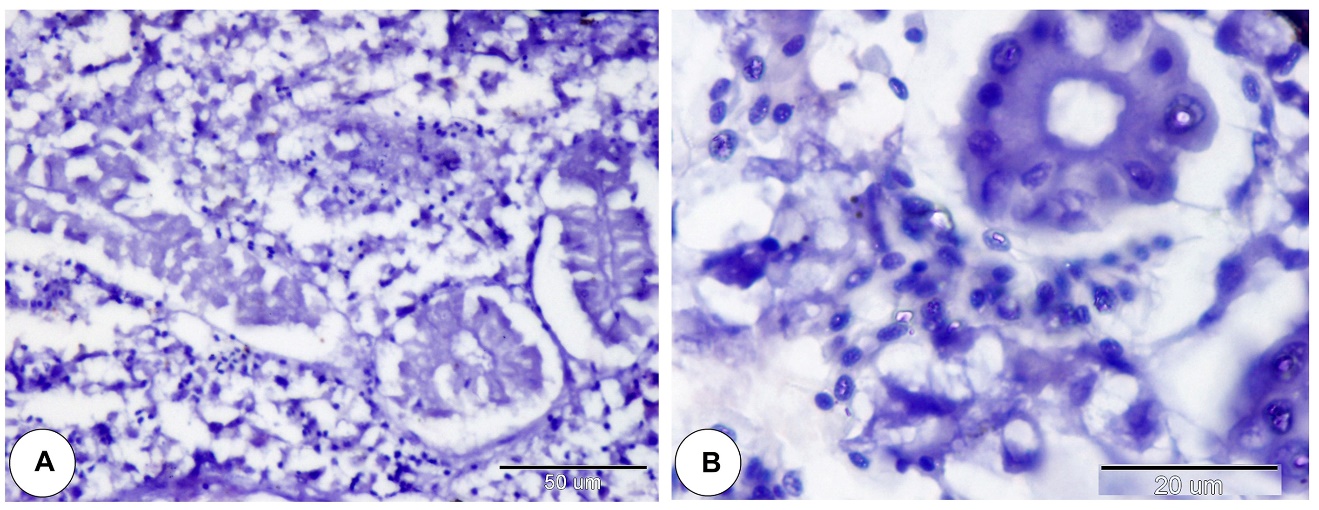


**Fig. S1. (A, B)** Negative control sections where The S100 protein primary antibody was omitted and instead, tissue specimens were incubated with buffer.
